# Periostin Links Mechanical Strain to Inflammation in Abdominal Aortic Aneurysm

**DOI:** 10.1371/journal.pone.0079753

**Published:** 2013-11-19

**Authors:** Osamu Yamashita, Koichi Yoshimura, Ayako Nagasawa, Koshiro Ueda, Noriyasu Morikage, Yasuhiro Ikeda, Kimikazu Hamano

**Affiliations:** 1 Department of Surgery and Clinical Science, Yamaguchi University Graduate School of Medicine, Ube, Japan; 2 Graduate School of Health and Welfare, Yamaguchi Prefectural University, Yamaguchi, Japan; 3 Department of Medicine and Clinical Science, Yamaguchi University Graduate School of Medicine, Ube, Japan; Brigham and Women's Hospital, Harvard Medical School, United States of America

## Abstract

**Aims:**

Abdominal aortic aneurysms (AAAs) are characterized by chronic inflammation, which contributes to the pathological remodeling of the extracellular matrix. Although mechanical stress has been suggested to promote inflammation in AAA, the molecular mechanism remains uncertain. Periostin is a matricellular protein known to respond to mechanical strain. The aim of this study was to elucidate the role of periostin in mechanotransduction in the pathogenesis of AAA.

**Methods and Results:**

We found significant increases in periostin protein levels in the walls of human AAA specimens. Tissue localization of periostin was associated with inflammatory cell infiltration and destruction of elastic fibers. We examined whether mechanical strain could stimulate periostin expression in cultured rat vascular smooth muscle cells. Cells subjected to 20% uniaxial cyclic strains showed significant increases in periostin protein expression, focal adhesion kinase (FAK) activation, and secretions of monocyte chemoattractant protein-1 (MCP-1) and the active form of matrix metalloproteinase (MMP)-2. These changes were largely abolished by a periostin-neutralizing antibody and by the FAK inhibitor, PF573228. Interestingly, inhibition of either periostin or FAK caused suppression of the other, indicating a positive feedback loop. In human AAA tissues in *ex vivo* culture, MCP-1 secretion was dramatically suppressed by PF573228. Moreover, *in vivo*, periaortic application of recombinant periostin in mice led to FAK activation and MCP-1 upregulation in the aortic walls, which resulted in marked cellular infiltration.

**Conclusion:**

Our findings indicated that periostin plays an important role in mechanotransduction that maintains inflammation via FAK activation in AAA.

## Introduction

Abdominal aortic aneurysm (AAA) is a common disease that causes segmental expansion and rupture of the aorta with a high mortality rate [1], [2]. Currently, therapeutic options for AAA are limited to open or endovascular surgical repair to prevent the catastrophic event of rupture [3]. The lack of nonsurgical treatment represents an unmet need, particularly in terms of pharmacotherapy [2], [4]. The development of biomarkers for AAA is also essential to produce clinically practical pharmacotherapy [2], [5]. It is generally accepted that an AAA is characterized by chronic inflammation and extracellular matrix degradation caused by proteolytic enzymes, such as matrix metalloproteinases (MMPs) [6], [7]. In addition, it was reported that successful endovascular aneurysm repair caused decreases in the size of the AAA and in plasma MMPs levels [8]. This suggested that mechanical stress is critical for maintaining disease activity in AAA, and that the signaling pathway stimulated by mechanical stress represents a therapeutic target. However, the molecular mechanisms that regulate mechanotransduction in AAA remain largely unknown.

Periostin is a matricellular protein, which belongs to the fasciclin I family. Periostin interacts with integrin molecules, including αvβ3 and αvβ5, on cell surfaces, which provides signals for tissue development and remodeling [9]. Previously, it was shown that periostin was upregulated by mechanical strain in cultured cells [10], [11] and also that periostin promoted the secretion of MMPs from cardiac cells [12]. These findings led to the hypothesis that periostin may play a role in the mechanotransduction involved in the pathogenesis of AAA. In the present study, we demonstrated for the first time that periostin mediated the inflammatory responses to mechanical strain in AAA in human tissue specimens, cultured vascular smooth muscle cells (VSMCs), and an *in vivo* mouse model.

## Methods

### Human aortic samples

We obtained abdominal aortic wall specimens from 42 patients with AAA that underwent open surgical repair. As controls, non-aneurysmal abdominal aortic wall specimens were obtained from 5 patients that underwent aortic surgery. The groups with and without AAA were not significantly different in age (74±1 vs. 68±5 years, *p* = 0.27), the proportion of males (76 vs. 60%, *p* = 0.43), or the prevalence of cigarette smoking (71 vs. 60%, *p* = 0.60). Also, patients with and without AAA were not significantly different in the prevalence of hypertension (73 vs. 60%, *p* = 0.56), diabetes mellitus (23 vs. 20%, *p* = 0.90), or dyslipidemia (23 vs. 20%, *p* = 0.72). The aortic tissue specimens were used for protein analyses by western blotting, immunohistochemistry, and *ex vivo* organ cultures, as previously described [13], [14]. All patients provided written informed consent, in accordance with the principles outlined in the Declaration of Helsinki. All experimental protocols with human specimens were approved by the Institutional Review Board at Yamaguchi University Hospital (#H24-26).

### Western blotting

Protein extraction and western blotting were performed as described previously [13], [14]. Briefly, equal amounts of sample proteins were loaded onto individual lanes in sodium dodecyl sulfate polyacrylamide gels, separated by electrophoresis, and transferred onto polyvinylidene difluoride membranes. Membranes were probed with antibodies against periostin (BioVendor, Brno, Czech Republic), monocyte chemoattractant protein-1 (MCP-1) (Cell Signaling Technology, Danvers, MA, USA), focal adhesion kinase (FAK) (Cell Signaling Technology), phosphorylated FAK (Tyr397) (Abcam, Cambridge, UK), extracellular signal-regulated kinase (ERK) (R&D Systems, Minneapolis, MN, USA), phosphorylated ERK (Promega, Madison, WI, USA), c-Jun N-terminal kinase (JNK) (Santa Cruz Biotechnology, Dallas, TX, USA), phosphorylated JNK (Promega), and glyceraldehyde 3-phosphate dehydrogenase (GAPDH) (Millipore, Billerica, MA, USA).

### Histological and immunohistochemical analyses

Paraffin-embedded sections were stained with hematoxylin/eosin (HE), Masson trichrome (MT), and elastica-van Gieson (EVG) stains for histological analysis. Sections were also probed with antibodies raised against appropriate antigens for immunohistochemistry, as described previously [13], [14]. We detected periostin, α-smooth muscle actin (α-SMA), MCP-1, and phosphorylated FAK (pFAK) by probing sections with anti-periostin antibody (BioVendor), anti-smooth muscle α-actin antibody (Sigma-Aldrich, St. Louis, MO, USA), anti-MCP-1 antibody (R&D Systems), and anti-pFAK antibody (Abcam), respectively.

### Gelatin zymography

Gelatin zymography was performed as described previously [13], [14]. The protein levels of MMP-2 and MMP-9 were determined by quantifying clear bands of the corresponding size.

### Enzyme-linked immunosorbent assay (ELISA)

The concentrations of MCP-1 in conditioned media were quantified by a sandwich enzyme immunoassay technique using the rat MCP-1 ELISA Kit (Thermo Scientific, Rockford, IL, USA) and the human MCP-1 ELISA Kit (R&D Systems), according to the manufacturer’s instructions.

### VSMC culture and mechanical strain experiments

Rat aortic VSMCs derived from the media of healthy rat aorta were purchased from Cell Applications, Inc (San Diego, CA, USA). VSMCs were maintained in Dulbecco's modified Eagle's medium (DMEM) with 10% fetal bovine serum. Before experiments, VSMCs were seeded in a laminin-coated silicon chamber and serum-starved for 24 h. Then, we applied cyclic, uniaxial strain to the VSMCs with a stretching system (STB-140-10) (STREX, Osaka, Japan). We divided the VSMCs into three groups of cells to test different amounts of strain. Each group received 2%, 5%, or 20% stretching along the long axis (elongation) at a frequency of 30 cycles/min for 48 h. For inhibition studies, 1 µg/ml of periostin-neutralizing antibody (R&D Systems) or 10 µM of FAK inhibitor (PF573228) (Tocris Bioscience, Bristol, UK) was added to the medium 2 h before stretching. In one experiment, VSMCs were stimulated with 1 µg/ml of recombinant mouse periostin (R&D Systems) for 48 h.

### *Ex vivo* cultures of human AAA specimens

The *ex vivo* organ culture was performed as described previously [13], [14]. Briefly, the AAA wall specimens were minced into approximately 1-mm thick. Equal wet weights of the minced tissue were placed in each well of 12-well plates and cultured with serum-free DMEM. For the inhibition study, 10 µM of FAK inhibitor (PF573228) (Tocris Bioscience) was added to the medium for 48 h.

### Animal experiments

For an observational study of changes in periostin levels during AAA development, we induced AAA in mice by periaortic application of 0.5 M CaCl_2_, as described previously [13], [15]. In the other study, we placed Gelfoam patches (3.5×2×2 mm) (Pfizer, New York, NY, USA) that contained 15 µg of recombinant mouse periostin (R&D Systems) in the periaortic space of mice for 7 days. Gelfoam patches with phosphate-buffered saline (PBS) served as the control. For both these studies, 7-week old, C57BL/6 male mice were anesthetized with an intraperitoneal injection of sodium pentobarbital (40 mg/kg) before undergoing a laparotomy. Anesthesia was monitored by periodic observation of respiration and the pain response. At the indicated time points, the experimental mice were sacrificed with an overdose of sodium pentobarbital (100 mg/kg, intraperitoneal injection). After a whole-body perfusion fixation with 4% paraformaldehyde in PBS at physiological pressure, the abdominal aortas were excised immediately for histological analysis. All experiments were performed in conformity with the Guide for the Care and Use of Laboratory Animals published by the United States National Institutes of Health, and the protocols were approved by the Yamaguchi University School of Medicine Animal Experiments Review Board (#31-068, #31-088).

### Statistical analysis

All data are expressed as mean ± standard error (SE). Statistical analyses were performed with the Student’s t-test, Mann-Whitney U test, or an analysis of variance (ANOVA), as appropriate. The post-test comparisons were performed with Bonferroni’s method.

## Results

### Expression of periostin in human AAAs

**Figure 1:**
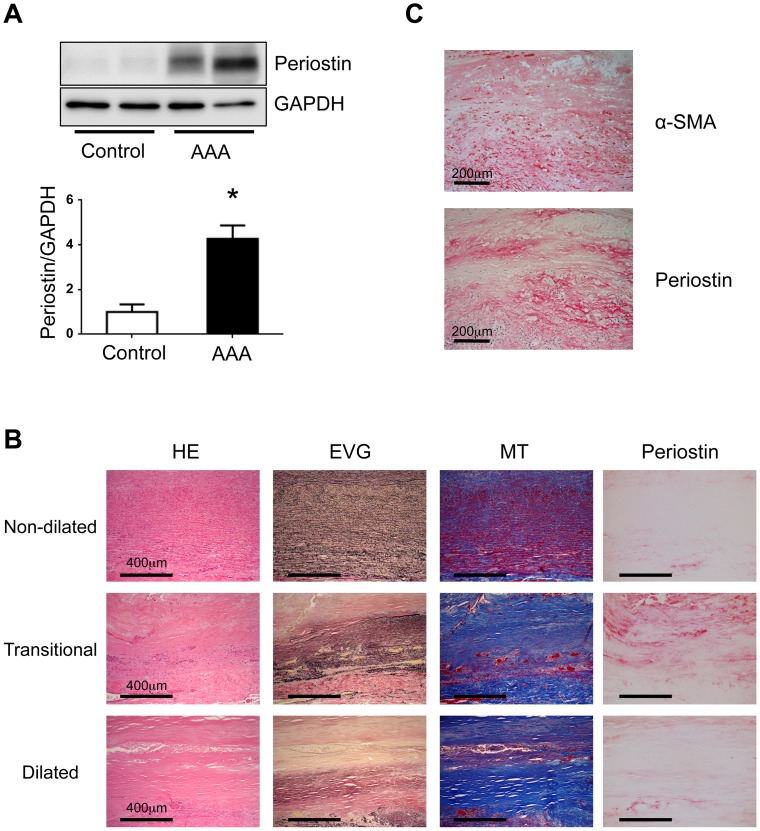
Expression of periostin in human AAA specimens. **A**: Protein samples were obtained from the walls of human AAA specimens (n = 42) and non-aneurysmal aortic walls (Control, n = 5). Representative western blot results for periostin are shown (top panel) with quantitative analyses (bottom panel). GAPDH served as an internal control. Data are mean ± SE. *p<0.05 compared to Control. **B**: Representative histological and immunohistochemical stains are shown for the walls of human AAA specimens. Different regions of the aorta include parts that are non-dilated (ND), transitional (T), and maximally dilated (MD). The luminal surface is oriented toward the top of each panel. Hematoxylin/eosin (HE), elastica van-Gieson (EVG), and Masson trichrome (MT) stains depict cell nuclei (blue-black), elastin network (black), and collagen fibers (blue), respectively. Localization of periostin is indicated by red staining. **C**: Localization of periostin and α-smooth muscle actin (α-SMA) is indicated by red staining.

First, we used western blotting to examine periostin protein levels in the walls of human AAA specimens. Compared to periostin levels in non-aneurysmal aortic walls (controls), periostin expression was significantly increased in the walls with AAA (**Fig. 1A**). However, we found no significant correlation between periostin expression and the diameter of AAA (data not shown); this lack of correlation was probably due to the heterogeneous pathology of AAA tissues. We also analyzed the localization of periostin within tissues to examine pathological changes that might be associated with increased periostin expression in AAA. All the typical pathological processes of AAA were included in longitudinal strips of the walls of human AAA specimens that extended from a non-dilated area through a transitional area to a dilated area [2], [15]. The non-dilated area showed preserved elastic lamellae with few inflammatory cells. The transitional area showed fragmented elastic fibers and severe cellular infiltration. The dilated area showed a marked loss of elastic lamellae and an increase in collagen fibers. Periostin expression was highest in the transitional area, where there was a gradual loss of elastic fibers and marked inflammatory cell infiltration (**Fig. 1B**). In addition, periostin and α-SMA, a marker for VSMCs, were mostly co-localized in human AAA wall specimens (**Fig. 1C**). Thus, our findings demonstrated that periostin was upregulated in human AAA wall specimens, particularly in the region of active inflammation.

### Temporal pattern of periostin expression during AAA progression in a mouse model

**Figure 2:**
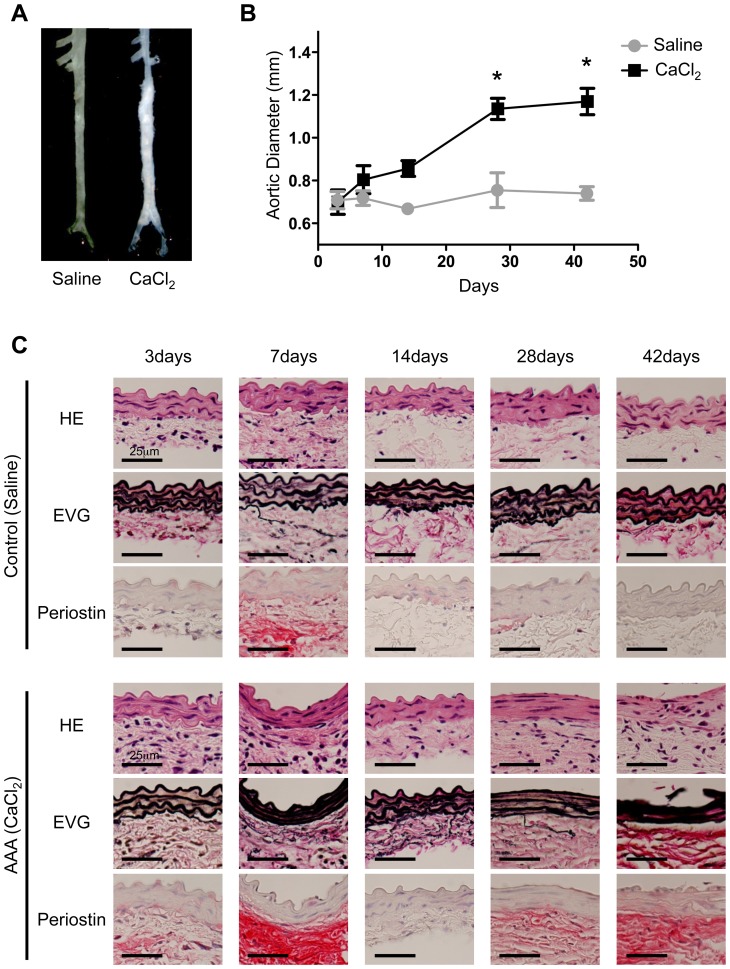
Temporal pattern of periostin expression during the development of abdominal aortic aneurysm (AAA) in a mouse model. A: Representative photographs show mouse abdominal aortas 42 days after periaortic application of saline (control) or CaCl_2_ (for AAA induction). B: Changes in the diameters of abdominal aortas are shown for the indicated time points after application of saline or CaCl_2_ (3 days, n = 3; 7 days, n = 4; 14 days, n = 4; 28 days, n = 3–4; 42 days, n = 7–8). Data are mean ± SE. *p<0.05 compared to saline controls. C: Representative images show aortic walls stained with hematoxylin/eosin (HE), elastica van-Gieson (EVG), or antibody against periostin at the indicated time points after application of saline or CaCl_2_.

Next, we analyzed a mouse model of AAA to evaluate periostin expression during the development and progression of AAA. The mouse model of AAA was created by periaortic application of CaCl_2_ in the infrarenal aorta; this caused chronic inflammation of the aortic wall and resulted in fusiform dilation of the aorta (**Fig. 2A**). After 28 and 42 days, the maximum diameter of the infrarenal aorta was significantly increased compared to controls (**Fig. 2B**). In the initial 7 days, periostin expression in the aorta was increased both in controls and in the AAA model mice; then, at 14 days, periostin returned to nearly basal levels. These data indicated that periostin was transiently induced after the surgical procedures and/or CaCl_2_ treatment. Interestingly, at 28 and 42 days, periostin expression once more increased, but only in the AAA model mice. At that same time, marked increases were noted in the aortic diameter, the inflammatory cell infiltration, and the destruction of elastic lamellae in AAA model mice (**Fig. 2C**). These results indicated that periostin was upregulated during the progression of AAA, particularly at the times that active inflammation was causing destruction of the extracellular matrix.

### Role of periostin in linking mechanical strain to inflammatory responses in VSMCs

**Figure 3:**
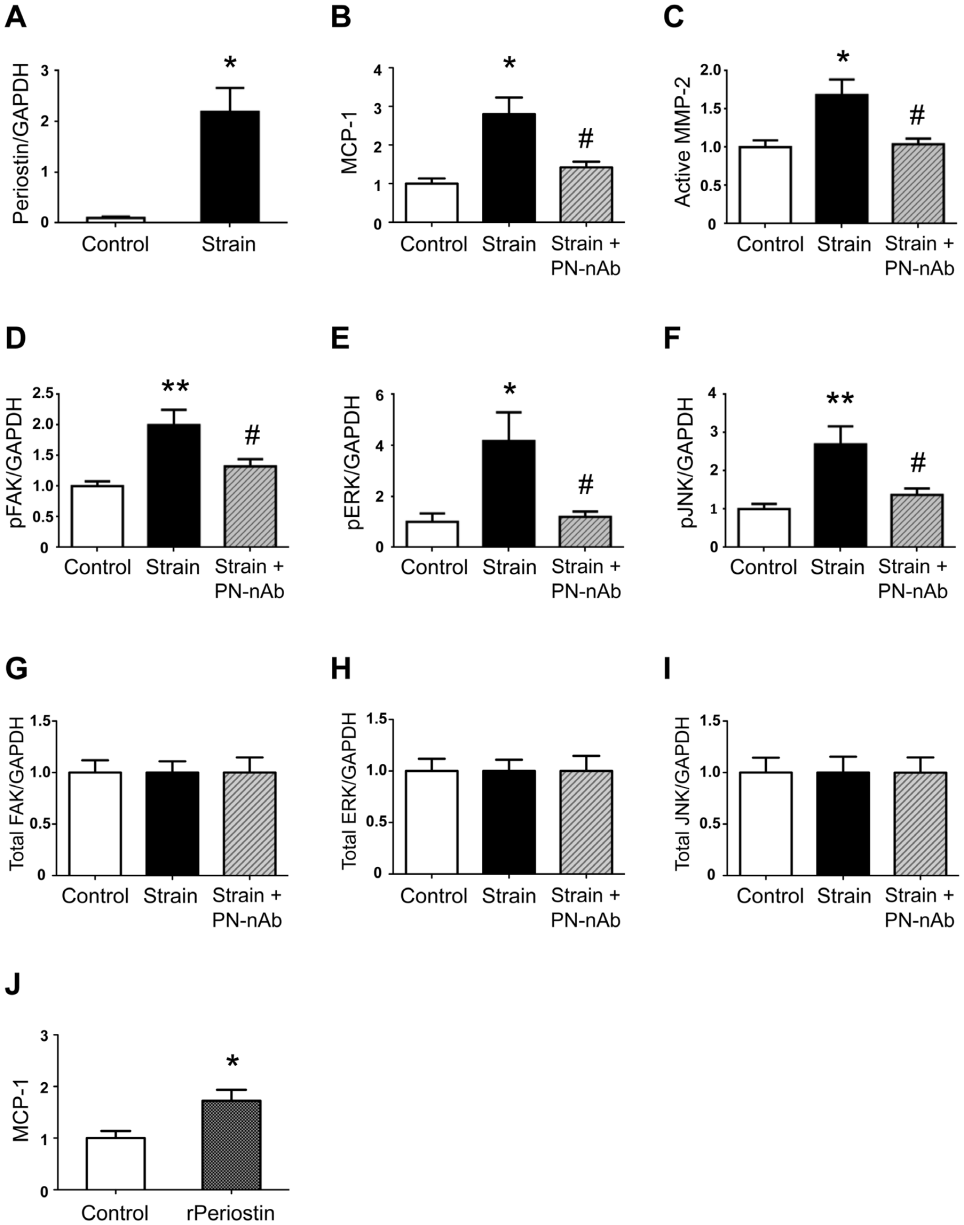
Role of periostin in linking mechanical strain to inflammatory responses in VSMCs. Cultured rat VSMCs treated with or without periostin-neutralizing antibody (PN-nAb, 1 µg/ml) were stimulated with cyclic uniaxial strain (20% elongation in length at a frequency of 30 cycles/min for 48 h, n = 6). Protein levels of periostin (**A**) and MCP-1 (**B**) in cell lysates were determined by western blotting. Levels of active MMP-2 in the conditioned media were determined by gelatin zymography (**C**). Levels of phosphorylated FAK (**D**), ERK (**E**) and JNK (**F**) in cell lysates were determined by western blotting. Levels of total FAK (**G**), ERK (**H**) and JNK (**I**) in cell lysates were determined by western blotting. GAPDH served as an internal control. Rat VSMCs were stimulated with recombinant periostin (rPeriostin; 1 µg/ml for 48 h; n = 4). Protein levels of MCP-1 in conditioned media were determined by ELISA (**J**). Data are mean ± SE. *p<0.05 and **p<0.01 compared to Control; #*p*<0.05 compared to VSMCs under mechanical strain.

To examine the role of periostin in mechanotransduction, we used cultured VSMCs as an experimental system. We chose VSMCs, because periostin expression had co-localized mainly with α-SMA in human AAA specimens. We applied cyclic, uniaxial strain at 30 cycles/min to VSMCs cultured on silicone rubber membranes. We found that 20% cyclic strain strikingly upregulated periostin protein expression in VSMCs (**Fig. 3A**), but not 2% or 5% strain (data not shown). The 20% cyclic strain also resulted in a significant increase in MCP-1 protein expression (**Fig. 3B**) and increased secretion of the active form of MMP-2 (**Fig. 3C**). Intriguingly, these increases in both MCP-1 and active MMP-2 induced by cyclic strain were completely abrogated when cells were pre-treated with periostin-neutralizing antibody (**Fig. 3B-C**). These data demonstrated an essential role for periostin in linking mechanical strain to upregulation of MCP-1 and active MMP-2 in VSMCs. Moreover, when VSMCs were treated with recombinant periostin (rPeriostin), secretion of MCP-1 was potently induced (**Fig. 3J**). Collectively, these results indicated that upregulation of periostin in response to mechanical strain was both necessary and sufficient for MCP-1 secretion from VSMCs. This effect could potentially lead to inflammatory cell infiltration.

### Role of periostin in mechanotransduction through FAK signaling in VSMCs

To understand the molecular mechanism that linked periostin to inflammatory responses, we focused on FAK; FAK has been reported to be activated by periostin [16], [17] and to stimulate MCP-1-mediated inflammatory cell recruitment [18]. Therefore, we stimulated VSMCs with 20% cyclic uniaxial strain and examined FAK activation by western blotting with an anti-pFAK antibody. We found that cyclic strain induced significant FAK activation without changing the total FAK levels in VSMCs, and this was abrogated by pre-treatment with a periostin-neutralizing antibody (**Fig. 3D, G**). These results indicated that periostin was essential for FAK activation induced by mechanical strain. Similarly, cyclic strain induced significant activation of ERK and JNK, two major signaling molecules that can regulate inflammation-associated gene expression in VSMCs. This effect was also abrogated by pre-treating with periostin-neutralizing antibody (**Fig. 3E-F, H-I**).

**Figure 4:**
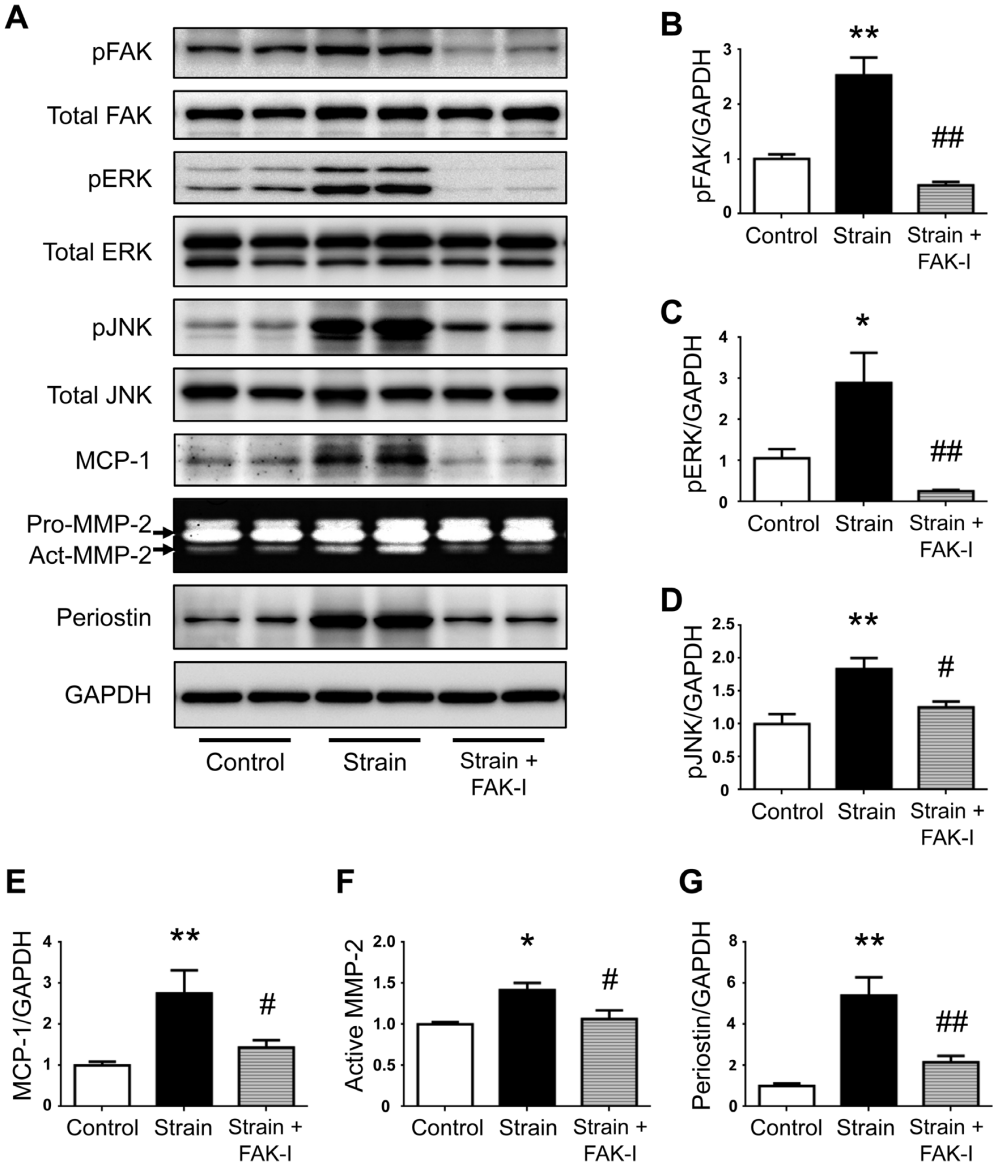
Role of periostin/FAK axis in mechanotransduction signaling pathway in VSMCs. Rat VSMCs were treated with or without of FAK inhibitor (PF573228, 10 µM, FAK-I) and stimulated with cyclic uniaxial strain (20% elongation in length at a frequency of 30 cycles/min for 48 h, n = 5). Levels of phosphorylated FAK (**A, B**), ERK (**A, C**) and JNK (**A, D**) in cell lysates were determined by western blotting. Protein levels of MCP-1 (**A, E**) and periostin (**A, G**) in cell lysates, and active MMP-2 (**A, F**) in conditioned media were determined by western blotting and gelatin zymography, respectively. GAPDH served as an internal control. Data are mean ± SE. *p<0.05 and **p<0.01 compared to Control; #*p*<0.05 and ##*p*<0.01 compared to VSMCs under mechanical strain.

We next used PF573228, a FAK inhibitor, to elucidate the molecular hierarchy in the network that links mechanical strain to inflammatory signaling through periostin. As reported previously, PF573228 entirely blocked activation of FAK by inhibiting its phosphorylation without changing total FAK levels (**Fig. 4A-B**). We found that PF573228 significantly prevented activation of ERK and JNK in response to mechanical strain applied to VSMCs (**Fig. 4A, C-D**). In addition, PF573228 blocked the mechanical strain-induced increases in both MCP-1 secretion and MMP-2 activity (**Fig. 4A, E-F**). Collectively, these findings indicated that the periostin/FAK axis was critical for activation of ERK and JNK and for increases in MCP-1 and active MMP-2 secretion in response to mechanical strain applied to VSMCs. Importantly, inhibition of FAK with PF573228 blocked the upregulation of periostin induced by mechanical strain (**Fig. 4A, G**), and inhibition of periostin with the neutralizing antibody blocked activation of FAK (**Fig. 3D**). Therefore, these findings revealed a vicious cycle between periostin and FAK that could amplify the inflammatory responses to mechanical strain in VSMCs.

### Role of periostin/FAK axis in sustaining inflammatory responses in human AAA tissues

**Figure 5:**
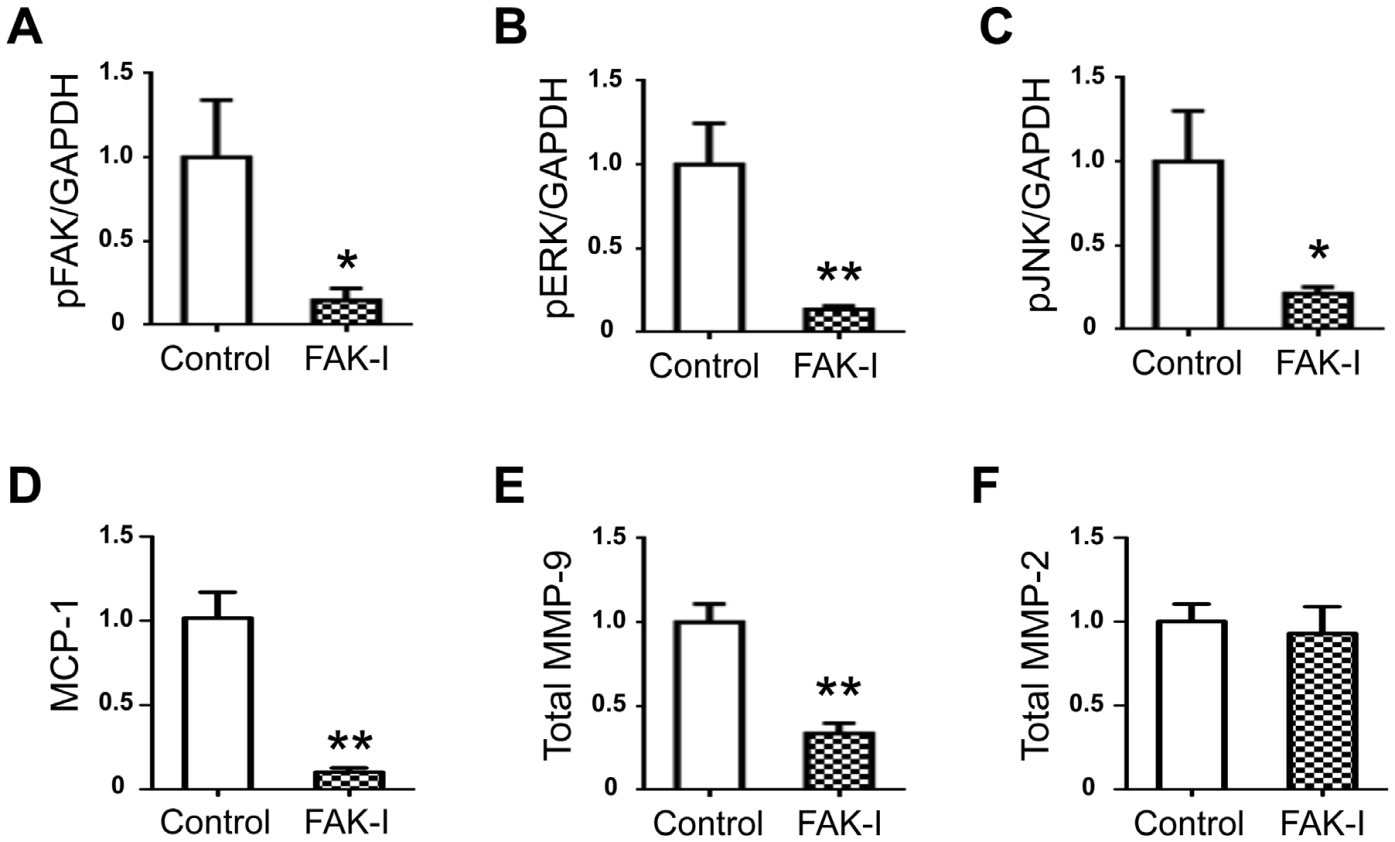
Role of periostin/FAK axis in sustaining inflammatory responses in human AAA tissues. Human AAA tissues were cut into small pieces and cultured with or without of FAK inhibitor (PF573228, 10 µM, FAK-I) for 48 h (n = 5). Levels of phosphorylated FAK (**A**), ERK (**B**) and JNK (**C**) in tissue lysates were determined by western blotting. GAPDH served as an internal control. Protein levels of MCP-1 (**D**) and MMPs (**E-F**) in conditioned media were determined by ELISA and gelatin zymography, respectively. Data are mean ± SE. *p<0.05 and **p<0.01 compared to Control.

We confirmed that the periostin/FAK axis played a role in the inflammatory responses in the walls of human AAA specimens in *ex vivo* cultures. Because there may be considerable differences in the pathophysiology of disease in humans and animal models, *ex vivo* cultures of human AAA tissues offered the great advantage of being able to analyze the response to an applied agent directly in human tissues [13], [19], [20]. We found that, in *ex vivo* cultures, human AAA tissues maintained considerable levels of FAK, ERK, and JNK activities and MCP-1 and MMP secretions during the experiments (**Fig. 5A-F**). We applied PF573228 to inhibit the periostin/FAK axis in human AAA tissues and confirmed that PF573228 permeated AAA tissues and blocked phosphorylation of FAK successfully (**Fig. 5A**). Additionally, PF573228 caused significant reductions in the activation levels of ERK and JNK in human AAA tissues (**Fig. 5B-C**). Also, PF573228 reduced the secretion of MCP-1 and MMP-9 from human AAA tissues (**Fig. 5D-E**). However, PF573228 did not affect the secretion levels of total MMP-2 (**Fig. 5F**); this result suggested that the viability of human AAA tissues in *ex vivo* cultures was preserved after treatment with PF573228. These data clearly demonstrated an essential role for the periostin/FAK axis in sustaining inflammatory responses in human AAA tissues.

### Role of periostin in inducing MCP-1-mediated inflammatory responses *in vivo*

**Figure 6:**
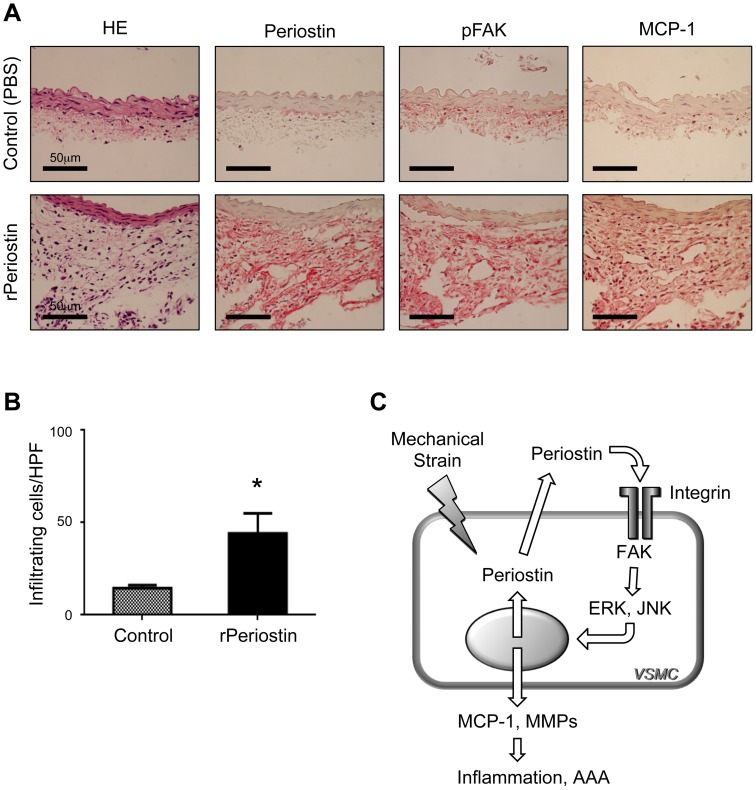
Role of periostin in inducing inflammatory responses *in vivo*. **A-B**: Gelfoam patches containing PBS (Control, n = 6) or recombinant periostin (rPeriostin, n = 7) were placed into periaortic spaces of mice for 7 days. Representative images show mouse aortic walls, stained with hematoxylin/eosin (HE) or with antibodies against periostin, phosphorylated FAK (pFAK), or MCP-1 (**A**). Infiltrating cells were counted in 5-10 high-power fields (**B**). Data are mean ± SE. *p<0.05 compared to Control. **C**: Schematic diagram represents the proposed vicious cycle of periostin upregulation driven by mechanical strain through activation of FAK, resulting in the maintenance of inflammation in AAA.

Based on the findings from our studies *in vitro* and *ex vivo*, we investigated whether periostin could sufficiently induce inflammatory responses in mouse aortas *in vivo*. To that end, we associated rPeriostin with Gelfoam, a biodegradable extracellular matrix preparation. This provided a local delivery system for rPeriostin, as reported previously [21]. We implanted Gelfoam patches loaded with rPeriostin or with buffer (control) into the periaortic spaces of mice, and then examined inflammatory responses after 7 days. Immunohistochemical analysis revealed that the periostin protein levels were significantly higher in rPeriostin-treated aortas; the antibodies may have reacted with both endogenous and exogenous periostin. Compared to control aortas, rPeriostin-treated aortas showed dramatically higher levels of FAK activation and MCP-1 expression, both in cells of the aortic wall and in cells in the periaortic space (**Fig. 6A**). Moreover, we found marked cellular infiltration into the rPeriostin-treated aortas (**Fig. 6A-B**). These results confirmed that periostin was sufficient for inducing *in vivo* inflammatory responses through FAK activation and MCP-1 upregulation.

## Discussion

The present study demonstrated that periostin was highly expressed in human AAA, principally in the regions of cellular infiltration and elastin degradation. Periostin was also upregulated during the progression of AAA in mouse, predominantly in the phase when persistent inflammation contributed to AAA expansion. Thus, periostin showed distinct spatial and temporal expression patterns. Periostin has advantageous properties for a biomarker, because it is deposited locally in inflammatory regions, and it is also released into the circulation. Serum levels of periostin have been recognized as a potential biomarker in patients with non-small cell lung carcinoma [22] and in patients with asthma [23]. Thus, periostin might serve as a useful marker for detecting active stages of AAA progression.

The walls of AAA vessels are theoretically exposed to abnormally high cyclic strain, according to the Laplace law. We demonstrated that periostin was upregulated with 20% cyclic strain in VSMCs, and it was persistently increased after enlargement of the aortic diameter in AAA model mice. These data suggested that, as the aorta expands, it is exposed to pathological cyclic strain; this strain persistently upregulates periostin expression, which then accelerates AAA progression. Thus, our results suggested that periostin plays a role in AAA progression, rather than AAA initiation. In addition, we found that, when rPeriostin was implanted with Gelfoam, it did not cause destruction of elastic lamellae or the initiation of AAA in mice after 42 days (O. Yamashita and K. Yoshimura, unpublished data). Those results indicated that a transient increase in periostin was insufficient to cause the development of AAA. This data supported the notion that periostin may not play a role in the initiation of AAA.

Importantly, we also elucidated the molecular mechanism by which periostin translated mechanical strain into biochemical signals that led to inflammatory responses in AAA. Previously, several lines of evidence had suggested that mechanical stress was critical for sustaining inflammatory responses and progression in AAA. For example, clinical cohort studies revealed a positive association between hypertension and the risk of AAA [24]. In addition, it was pointed out that exclusion of AAA from systemic pressure in an endovascular aneurysm repair could lead to a reduction in the size of the AAA [25], [26]. It was also reported that successful endovascular aneurysm repair caused decreases in the levels of serum interleukin-1α [27] and plasma MMP-9 [8], [28]. Together, those findings implied that hemodynamic stress could contribute to aneurysm progression, both by direct physical force and by accelerating molecular biological processes. However, the molecular basis of these effects was not identified previously. To address this challenge, we examined the molecular events in VSMCs in response to cyclic strain. VSMCs are the major cells for monitoring the mechanical environment in the aortic wall [29]. Hemodynamic forces primarily include cyclic and shear stresses. The cyclic, circumferential strain, which acts perpendicular to the aortic wall, causes outward stretching of VSMCs. Fluid shear stress is the frictional force generated as blood drags against the vascular endothelial cells in the aortic wall [30]. Notably, given the same blood pressure, vessels affected by AAA will experience higher cyclic strain than normal-sized aortic vessels, according to the Laplace law. Hence, in this study, we applied supraphysiological levels of cyclic strain to VSMCs. We used 20% cyclic strain, based on previously published studies, which showed that 15% to 20% strain could be considered pathologically-elevated stretching [31]–[33]. On the other hand, a number of studies have used 5% to 10% cyclic strain to represent physiologically-relevant stretch conditions [33]–[37]. Those lower strain levels were presumably based on the observation that the maximum stretch in the human aorta during the cardiac cycle was approximately 10% under normotensive conditions [38]. Reported *in vivo* levels of aortic cyclic strain have varied widely, ranging from nearly 2% to around 20% [39]–[41]. This variation was probably due to differences in the methods of measurement, the ages of subjects, and the presence or absence of pathological changes. As suggested previously, pathological cyclic strain (>15%) leads to VSMC maladaptation, with enhanced growth and secretion, but physiological levels of cyclic strain maintain healthy VSMCs in a quiescent state [34], [37], [42]. These findings lend support to our use of 20% cyclic strain in the present study to represent pathological cyclic strain.

Crucially, our findings demonstrated for the first time that the periostin/FAK axis played a key role in maintaining and amplifying the inflammatory responses to mechanical strain in AAA (**Fig. 6C**). It was previously reported that mechanical strain caused an increase in periostin mRNA levels in Lewis lung cancer cells [10] and in periodontal ligament fibroblasts [11]. In addition, periostin was shown to be essential for activation of FAK in VSMCs [16] and in cardiac cells [17]. More recently, it was reported that mechanical stress, though not a cyclic strain, caused FAK activation and promoted MCP-1 secretion through the FAK/ERK pathway in the pathological mechanisms of skin fibrosis [18]. In addition, MMP-2 and MMP-9 were reported to be upregulated by periostin in cardiac cells [12], and also by FAK activation in a fibroblast cell line [43]. Here, we integrated these findings and successfully demonstrated the involvement of the periostin/FAK axis in mechanotransduction in VSMCs. Surprisingly, we also discovered a vicious cycle between periostin production and FAK activation in VSMCs. We found that inhibition of either periostin or FAK resulted in suppression of both proteins. In human AAA tissues, we observed upregulation of periostin and significant activation of FAK. We also showed that inhibition of FAK dramatically decreased the activation of JNK. We previously found that secretion of periostin was significantly diminished with a JNK inhibitor, SP600125, in *ex vivo* human AAA tissue cultures (K. Yoshimura, unpublished data). Taken together, these findings strongly suggested that a vicious cycle among periostin, FAK, and JNK could amplify the inflammatory responses to mechanical strain in human AAA. Thus, our results suggested that relentless pathological strain on AAA walls leads to continuous activation of the periostin/FAK axis, thereby causing sustained upregulation of MCP-1 and MMPs, and finally resulting in progression of AAA. Indeed, our results showed that application of periostin in mouse aortic walls activated FAK and upregulated MCP-1, which resulted in cellular infiltration. It was previously known that MCP-1 and MMPs were highly upregulated in the walls of human AAA specimens [13], [44]; in addition, these molecules also played critical roles in inflammatory cell infiltration and extracellular matrix degradation during the development of AAA in mice [45], [46]. We previously reported that JNK played an essential role in abnormal extracellular matrix metabolism and disease progression in AAA. We also showed that inhibition of JNK prevented the development of AAA and could also cause regression of established AAA in mice [13]. Hence, JNK is a key molecule in the pathogenesis of AAA. In the current work, we showed that JNK was also involved in the vicious cycle of periostin upregulation, driven by mechanical strain, during the progression of AAA.

In conclusion, this study provided new insights into the underlying mechanisms that link periostin to mechanical strain and inflammation in the progression of AAA. Our findings revealed a novel vicious cycle, which is driven by mechanical strain, and leads to the amplification and maintenance of inflammatory responses. Thus, periostin may represent both a clinical biomarker for disease activity in AAA and a therapeutic target for patients with AAA.
